# Egr-1 mediates low-dose arecoline induced human oral mucosa fibroblast proliferation via transactivation of Wnt5a expression

**DOI:** 10.1186/s12860-020-00325-7

**Published:** 2020-11-10

**Authors:** Qiang Chen, Jiuyang Jiao, Youyuan Wang, Zhihui Mai, Jing Ren, Sijie He, Xiaolan Li, Zheng Chen

**Affiliations:** 1 Department of Stomatology, the Third Affiliated Hospital of Sun Yat-sen University, No.600 Tianhe road, Guangzhou, 510630 China; 2 Department of Oral & Maxillofacial Surgery & Guangdong Provincial Key Laboratory of Malignant Tumor Epigenetics and Gene Regulation, Sun Yat-sen Memorial Hospital, Sun Yat-sen University, Guangzhou, China; 3 The fourth people’s hospital of Nanhai district of Foshan city, Foshan, China; 4 Guanghua School of stomatology & hospital of stomatology, Guangdong province key laboratory of stomatology, Sun Yat-sen University, Guangzhou, China

**Keywords:** Arecoline, Wnt5a, Egr-1, Fibrosis, Oral submucous fibroblast

## Abstract

**Background:**

Arecoline is an alkaloid natural product found in the areca nut that can induce oral submucous fibrosis and subsequent development of cancer. However, numerous studies have shown that arecoline may inhibit fibroblast proliferation and prevent collagen synthesis.

**Results:**

High doses of arecoline (> 32 μg/ml) could inhibit human oral fibroblast proliferation, while low doses of arecoline (< 16 μg/ml) could promote the proliferation of human oral fibroblasts. Wnt5a was found to be both sufficient and necessary for the promotion of fibroblast proliferation. Egr-1 could mediate the expression of Wnt5a in fibroblasts, while NF-κB, FOXO1, Smad2, and Smad3 did not. Treatment with siRNAs specific to Egr-1, Egr inhibitors, or Wnt5a antibody treatment could all inhibit arecoline-induced Wnt5a upregulation and fibroblast proliferation.

**Conclusions:**

Egr-1 mediates the effect of low dose arecoline treatment on human oral mucosa fibroblast proliferation by transactivating the expression of Wnt5a. Therefore, Egr inhibitors and Wnt5a antibodies are potential therapies for treatment of oral submucosal fibrosis and oral cancer.

## Background

Arecoline, the main alkaloid compound found in the Areca nut, is the fourth most commonly consumed psychoactive substance in the world, following only ethanol, nicotine, and caffeine in prevalence [1, 2]. Areca nuts are particularly prevalent in Asian countries such as China, Taiwan, and India, and their popularity continues to expand [3, 4]. Chewing areca nuts is closely related to development of a variety of oral diseases, including oral submucous fibrosis (OSF), oral leukoplakia, and oral cancer [4–6]. OSF used to be reported mainly in Southeast Asia, but it is now also found in the Asian immigrant populations of Britain and America and as such has become a global health problem [7]. The main pathological manifestation of OSF is abnormal accumulation of collagen in the lamina propria under the oral mucosa [8]. Following development of OSF, 3–19% of patients may develop cancer, and this probability increases yearly [9]. The habit of chewing areca nuts is considered to be the most likely factor for the occurrence and malignancy of OSF [10]. In 2003, the World Health Organization listed areca nuts as a primary carcinogen. However, the role of arecoline in the pathogenesis of oral disease is still controversial. Many studies have found that arecoline can inhibit cell proliferation and migration, stimulate cell differentiation, and induce apoptosis [11–14], but some studies have also observed that arecoline can promote the proliferation and migration of some cell types [15, 16].

Wnt family members are secreted glycoproteins that are highly conserved and play a key role in the regulation of fibroblast proliferation and tissue fibrosis [17–20]. For example, it has been reported that the pro-fibrotic Wnt1/βcatenin injury response is essential for preserving cardiac function following acute ischemic cardiac injury [21]. Other studies have found that Wnt3a could induce myofibroblast differentiation by upregulating TGF-β signaling through SMAD2 in a β-catenin-dependent manner [22]. Furthermore, Vuga and Kaminski et al. observed that Wnt5a was a regulator of fibroblast proliferation and resistance to apoptosis [23]. Therefore, Wnt family members may serve as ideal molecular targets for controlling fibroblast proliferation. However, the relationship between arecoline exposure and Wnt, as well as the transcription factors that control Wnt expression in oral fibroblasts, remain unclear.

In this study, we found that low-dose arecoline treatment promoted human oral fibroblast proliferation and that Egr-1 upregulated Wnt5a expression to mediate the proliferative effect of arecoline treatment. Collectively, these findings establish Egr-1 and Wnt5a as new potential therapeutic targets for treatment of OSF caused by chewing areca nuts.

## Results

### Low-dose arecoline treatment induces fibroblast proliferation

Previous studies have shown that arecoline is cytotoxic to oral fibroblasts at concentrations of 50 μg/ml or greater [12]. Chewing areca nuts is known to increase the risk of oral cancer and OSF. The level of arecoline present in the saliva during areca nut chewing has been found to be around 0.1 μg/ml, and has been found to increase to about 0.3 μg/ml after chewing [24]; these concentrations are much lower than the concentration of arecoline used in most experiments.

**Fig. 1:**
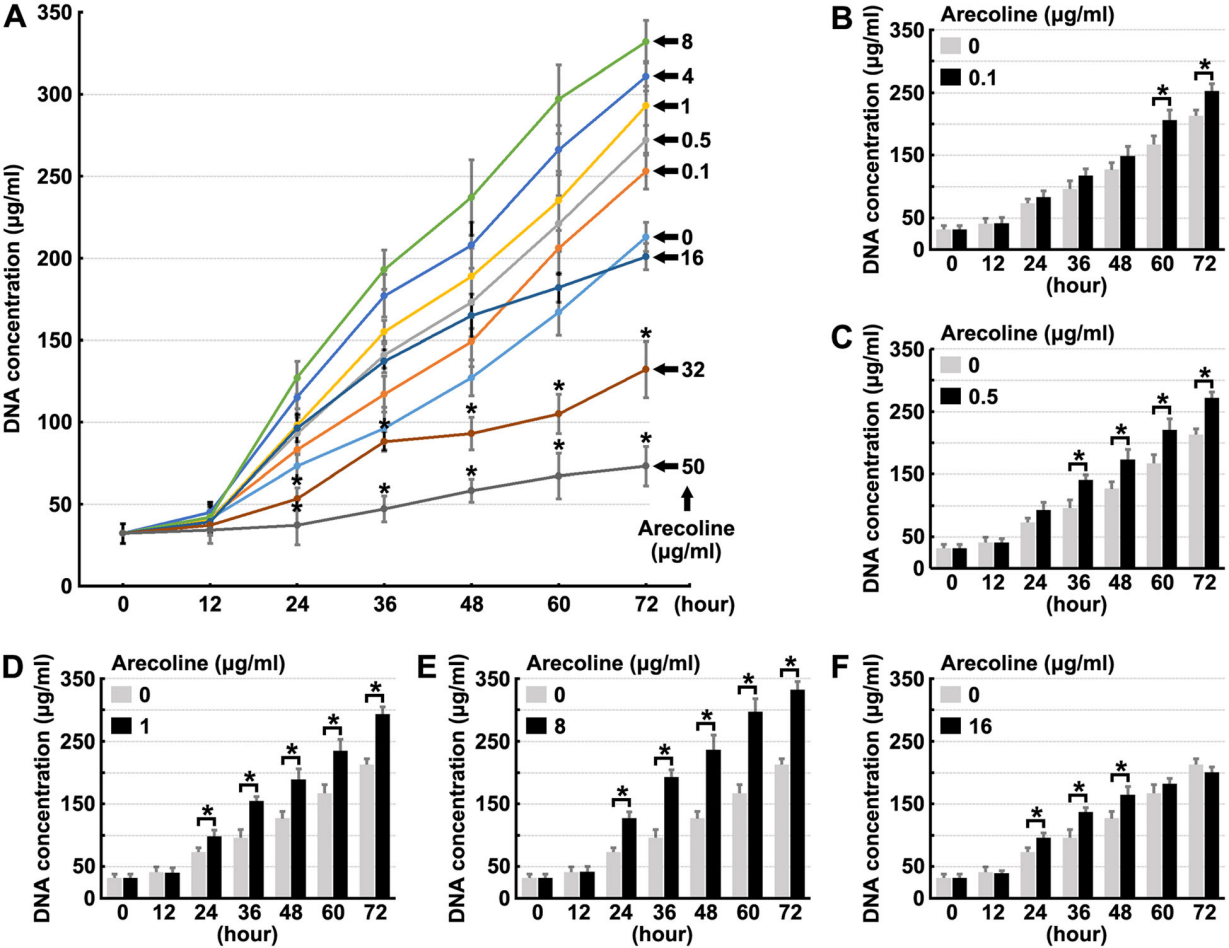
Low dose Arecoline induces the proliferation of fibroblasts. Human oral fibroblasts were treated with different dose Arecoline for indicated times, then cell proliferation rate was quantified. * denotes *p* < 0.05

In the present study, we intend to test the effect of different doses of arecoline (0.1, 0.5, 1, 4, 8, 16, 32 or 50 μg/ml) on human oral fibroblast proliferation. In these experiments, half of the medium was refreshed every 24 h. We confirmed that high doses of arecoline (32 μg/ml and 50 μg/ml) could inhibit fibroblast proliferation (Fig. 1a and Fig. S1). Our results also indicated that arecoline treatment promoted fibroblast proliferation at concentrations ranging from 0.1–16 μg/ml (Fig. 1b-f and Fig. S1), and the maximum effect on proliferation was observed at 8 μg/ml (Fig. 1e and Fig. S1). These results demonstrated that low doses of arecoline could promote human oral fibroblast proliferation.

### Arecoline treatment promotes fibroblast proliferation by inducing Wnt5a expression

**Fig. 2:**
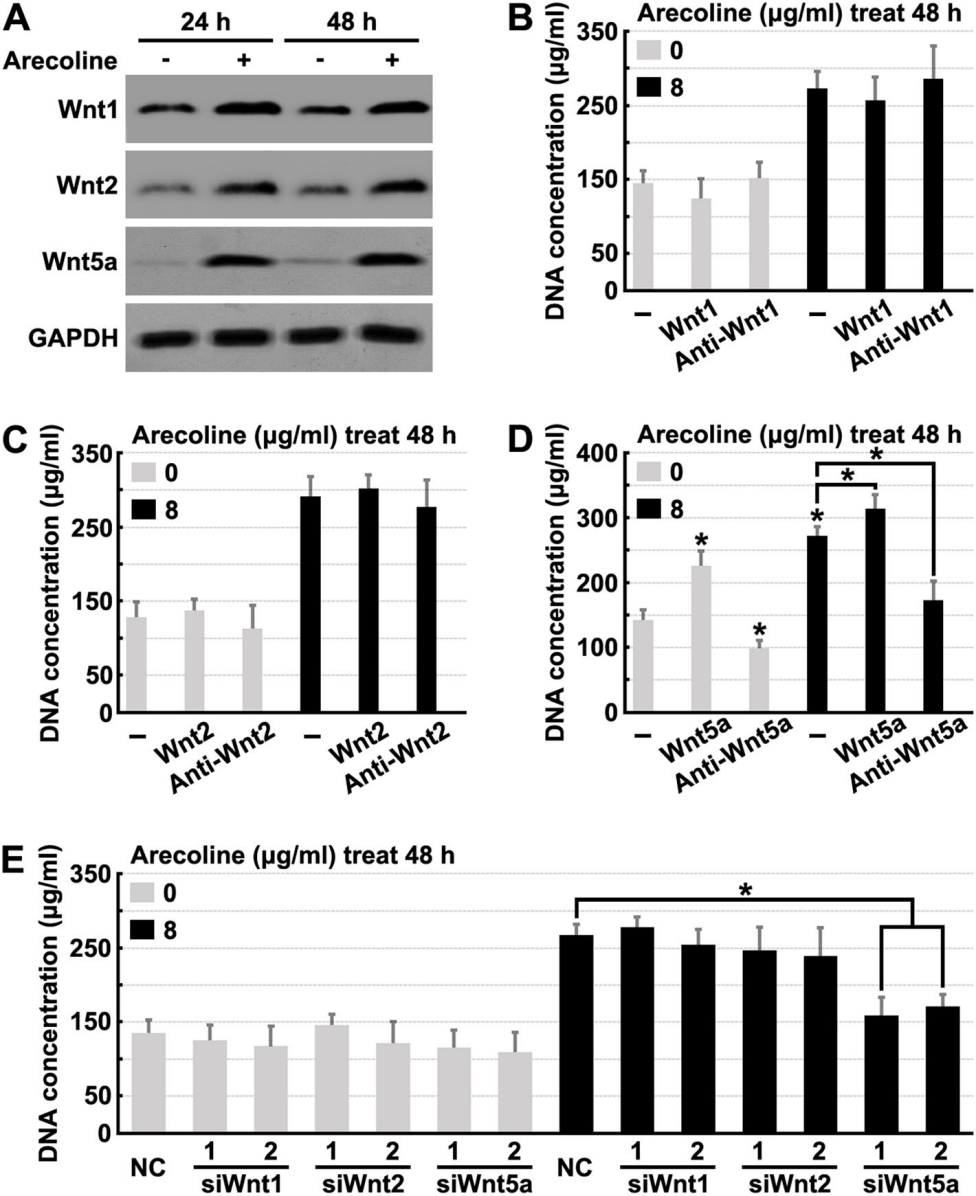
Arecoline promotes fibroblasts proliferation by inducing Wnt5a expression. **a** Human oral fibroblasts were treated with or without 8 μg/ml Arecoline for indicated times, then cell lysates were analyzed by Western blotting using indicated antibodies. **b-d** Human oral fibroblasts were treated with 8 μg/ml Arecoline, recombinant Wnt1, Wnt2 or Wnt5a protein and Wnt1, Wnt2 or Wnt5a antibody for 48 h, then cell proliferation rate was quantified. **e** Human oral fibroblasts were treated with 8 μg/ml Arecoline, Wnt1, Wnt2 or Wnt5a sepecific siRNAs for 48 h, then cell proliferation rate was quantified. * denotes *p* < 0.05

Earlier reports have shown that activation of Wnt/β-catenin signaling may promote fibroblast proliferation by regulating the expression of Wnt1, Wnt2, Wnt3a, or Wnt5a [23]. To determine whether other Wnt isoforms play a role in regulating human oral fibroblast proliferation, the mRNA expression levels of all 19 Wnt gene family members in human oral fibroblasts exposed to 8 μg/ml arecoline for 24 h were analyzed by RT-PCR. As illustrated in Table 1, arecoline treatment altered the transcription of ten Wnts (Wnt1, 2, 3a, 5a, 5b, 8b, 10a, 10b, 11, and 16). Of those, Wnt3a, Wnt5b, Wnt8b, Wnt10a, Wnt10b, Wnt11, and Wnt16 were expressed only at very low levels in both the control and treatment groups, whereas Wnt1, Wnt2, and Wnt5a were expressed at higher levels. We then analyzed the protein expression levels of Wnt1, Wnt2, and Wnt5a in a time course experiment in fibroblasts treated with 8 μg/ml arecoline. As expected, arecoline treatment significantly promoted the expression of the Wnt1, Wnt2, and Wnt5a proteins (Fig. 2a).

**Table 1:** Normalized mRNA expression of Wnts

Gene Name	Control		Arecoline 8 μg/ml for 24 h	
	Relative Fold	CT value	Relative Fold	CT value
Wnt1^*^	1	27.53 ± 0.86	1.92 ± 0.18	26.71 ± 0.69
Wnt2^*^	1	29.26 ± 0.29	2.25 ± 0.21	28.12 ± 0.54
Wnt2b	1	33.09 ± 1.37	1.12 ± 0.33	32.84 ± 1.71
Wnt3	1	30.41 ± 0.84	0.92 ± 0.22	31.38 ± 1.25
Wnt3a^*^	1	36.17 ± 1.26	1.42 ± 0.27	35.67 ± 0.85
Wnt4	1	21.71 ± 0.42	0.87 ± 0.21	22.32 ± 0.62
Wnt5a^*^	1	23.59 ± 0.31	2.57 ± 0.64	21.89 ± 0.78
Wnt5b^*^	1	34.12 ± 1.73	1.63 ± 0.22	32.25 ± 1.47
Wnt6	1	29.35 ± 0.68	0.94 ± 0.15	28.46 ± 0.33
Wnt7a	1	37.31 ± 1.79	1.07 ± 0.26	36.77 ± 1.36
Wnt7b	1	31.64 ± 0.52	0.95 ± 0.25	32.53 ± 0.90
Wnt8a	1	28.03 ± 1.15	1.17 ± 0.39	27.41 ± 1.17
Wnt8b^*^	1	35.52 ± 1.27	1.78 ± 0.32	34.93 ± 0.83
Wnt9a	1	26.47 ± 1.07	0.89 ± 0.17	27.05 ± 0.79
Wnt9b	1	28.02 ± 1.33	0.96 ± 0.11	28.62 ± 0.41
Wnt10a^*^	1	35.74 ± 2.28	1.44 ± 0.27	34.62 ± 1.49
Wnt10b^*^	1	36.17 ± 1.41	1.75 ± 0.48	34.91 ± 1.58
Wnt11^*^	1	34.86 ± 1.73	2.63 ± 1.39	33.17 ± 1.37
Wnt16^*^	1	36.77 ± 1.03	1.81 ± 0.42	35.82 ± 0.73

The values represent the mean ± S.E. of three independent experiments. ^*^P < 0.05

To determine if Wnt1, Wnt2, or Wnt5a were required for the effect of arecoline treatment on fibroblast proliferation, human oral fibroblasts were treated with either recombinant Wnt1, Wnt2, or Wnt5a protein and Wnt1, Wnt2, or Wnt5a. The results of these experiments revealed that fibroblast proliferation was not affected by Wnt1 or Wnt2 protein or antibody (Fig. 2b, c and Fig. S2a, b); however, treatment with recombinant Wnt5a protein was found to increase fibroblast proliferation (Fig. 2d and Fig. S2c). Furthermore, the effect of Arecoline treatment on fibroblast proliferation was inhibited by treatment with Wnt5a antibody (Fig. 2d and Fig. S2c). We also found that siRNAs specific to Wnt5a inhibited arecoline-induced fibroblast proliferation, while fibroblast proliferation was not affected by Wnt1 or Wnt2 siRNAs (Fig. 2e and Fig. S2d). Together, these results demonstrated that Wnt5a mediated the effect of arecoline treatment on fibroblast proliferation.

### Egr-1 is necessary for the expression of Wnt5a

**Fig. 3:**
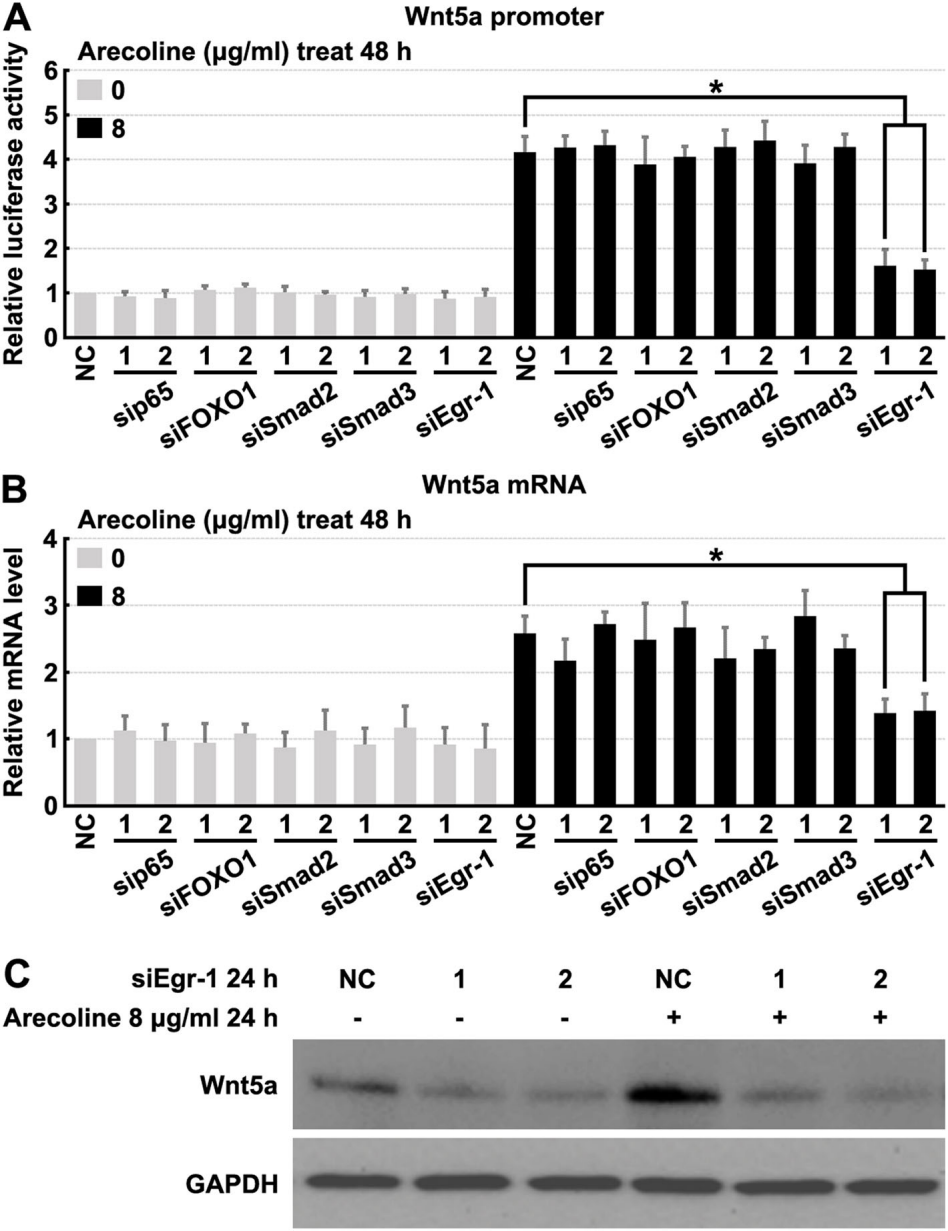
Egr-1 is necessary for the expression of Wnt5a. **a** Human oral fibroblasts were transfected with Wnt5a promoter luciferase reporter plasmids, treated with 8 μg/ml Arecoline and p65, Smad2, Smad3 or Egr-1 siRNAs for 48 h, then luciferase reporter assays were performed to detect the activity of Wnt5a promoter. **b** Human oral fibroblasts were treated with 8 μg/ml Arecoline and p65, Smad2, Smad3 or Egr-1 siRNAs for 48 h, then RT-PCR assays were performed to detect the mRNA levels of Wnt5a. **c** Human oral fibroblasts were treated with 8 μg/ml Arecoline and Egr-1 siRNAs for 24 h, then cell lysates were analyzed by Western blotting using indicated antibodies. * denotes *p* < 0.05

Previous studies have identified many transcription factor binding sites in the human Wnt5a promoter, such as NF-κB, FOXO1, Smad2, Smad3, and Egr-1 [25, 26]. To identify signaling mechanisms regulating Wnt5a expression, siRNAs specific to NF-κB p65, FOXO1, Smad2, Smad3, or Egr-1 were tested. The results of these experiments revealed that NF-κB p65, FOXO1, Smad2, and Smad3 siRNAs could not affect the promoter activity of Wnt5a or the expression of Wnt5a (Fig. 3a and b), indicating an NF-κB p65, FOXO1, Smad2, and Smad3-independent regulation of Wnt5a expression.

To determine whether Egr-1 was involved in Wnt5a regulation, fibroblasts were transfected with Egr-1 siRNAs. Arecoline-induced Wnt5a expression was effectively blocked by Egr-1 siRNAs (Fig. 3a and c), confirming that Egr-1 is involved in Wnt5a regulation in fibroblasts. Treatment with Egr-1 siRNAs significantly suppressed Wnt5a protein expression (Fig. 3c). Therefore, we concluded that Egr-1 is essential for transcriptional induction of Wnt5a expression in human oral fibroblasts.

### Inhibition of Egr activity prevents arecoline-induced fibroblast proliferation

**Fig. 4:**
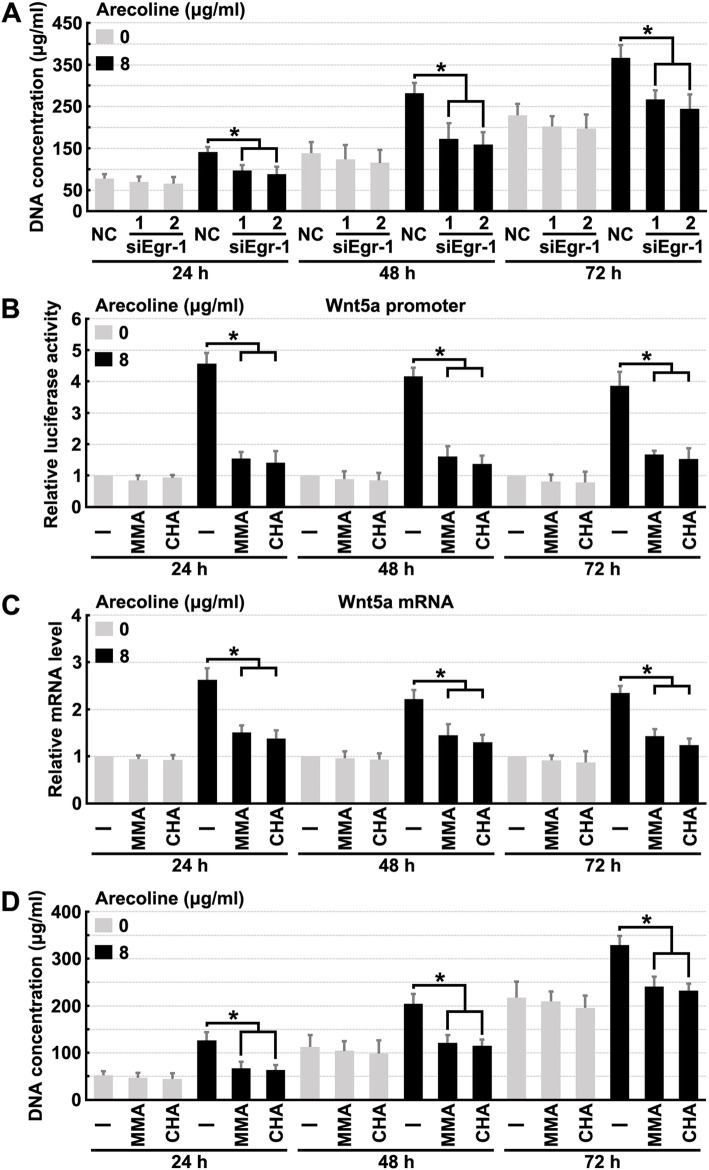
Inhibition of Egr activity prevents Arecoline induced fibroblast proliferation. **a** Human oral fibroblasts were treated with 8 μg/ml Arecoline, Wnt1, Wnt2 or Wnt5a sepecific siRNAs for indicated times, then cell proliferation rate was quantified. **b** Human oral fibroblasts were transfected with Wnt5a promoter luciferase reporter plasmids, treated with 8 μg/ml Arecoline, 2 μM Mithramycin A (MMA) or 1 μM Chromomycin A3 (CHA) for indicated times, then luciferase reporter assays were performed to detect the activity of Wnt5a promoter. **c-d** Human oral fibroblasts were treated with 8 μg/ml Arecoline, 2 μM MMA or 1 μM CHA for indicated times, then RT-PCR assays were performed to detect the mRNA levels of Wnt5a (**c**) or cell proliferation rate was quantified (**d**). * denotes *p* < 0.05

We next assessed the role of Egr-1 in modulating fibroblast proliferation. The results of these experiments showed that Egr-1 knockdown inhibited the effect of arecoline treatment on fibroblast proliferation (Fig. 4a and Fig. S3a). Furthermore, mithramycin A (MMA) and chromomycin A3 (CHA) were used to treat fibroblasts (MMA and CHA repress transcription by selectively displacing GC-rich DNA binding transcription factors, such as Egr-1 [27, 28]). The results of these experiments revealed that MMA or CHA treatment blocked arecoline-induced Wnt5a upregulation and promotion of fibroblast proliferation (Fig. 4b, c, d and Fig. S3b). These results indicated that the expression and activity of Egr-1 were required for driving the effect of arecoline treatment on fibroblast proliferation.

## Discussion

Areca nuts contain a variety of substances, including alkaloids, polyphenols and nitrosamines. Among them, the alkaloids compounds are arecoline, tetrahydronicotinic acid, and others [29].. Arecoline is the main carcinogenic compound found in areca nuts and prolonged exposure to arecoline can induce OSF and development of oral cancer. However, many studies have shown that arecoline treatment inhibits cell growth, cell proliferation, and collagen synthesis in human oral fibroblasts in a dose-dependent manner [11, 12, 30]. Chang et al. found that arecoline was cytotoxic to human oral fibroblasts at concentrations greater 50 μg/ml due to depletion of intracellular thiols and inhibition of mitochondrial activity [12, 30]; Jeng et al. found that arecoline treatment inhibited the migration, attachment, spreading, growth, and collagen synthesis of human oral fibroblasts at concentrations of 0.4 mM (62 μg/ml) and 1 mM (155 μg/ml) [11]. Venkatesh et al. found that arecoline levels in the saliva were about 0.1 μg/ml to about 0.3 μg/ml after chewing commercially available areca nuts [24], which is much lower than the concentration of arecoline used in most experiments. In this study, we found that low doses of arecoline could promote the proliferation of human oral fibroblasts. In accordance with our findings, Xia et al. found that a relative low dose (20 μg/ml) arecoline treatment could increase oral fibroblast collagen production [31]. These findings indicate that more studies should focus on the effects of low-dose arecoline in the pathogenesis of oral diseases.

Recently, the role of the Wnt/β-catenin pathway has been identified as one of the central mechanisms behind pulmonary, hepatic, renal, and cardiac fibrosis [20, 32–34]. Among the numerous Wnt family members, Wnt5a has been found to be closely related to fibrosis. Vuga et al. found that Wnt5a played a role in fibroblast expansion and survival in idiopathic pulmonary fibrosis and other fibrotic interstitial lung diseases that exhibit typical interstitial pneumonia histological patterns [23]. Villar et al. suggested that the Wnt/β-catenin signaling pathway is activated very early in sepsis-induced acute respiratory distress syndrome and could play an important role in lung repair and fibrosis [35]. Abraityte et al. found that Wnt5a is elevated in the serum and myocardium of heart failure (HF) patients and this elevation promoted myocardial inflammation and fibrosis [36]. Martin-Medina et al. found that Wnt5a was secreted in extracellular vesicles in lung fibrosis and induced by TGF-β signaling in primary human lung fibroblasts [37]. We screened the expression of Wnt family members in oral fibroblasts after arecoline treatment and further determined their basic functions in this system. Our experiments found that Wnt5a played a role in the effect of low dose arecoline treatment on human oral fibroblast proliferation. This finding suggests that the treatment of OSF may share some similarity to the treatment strategies utilized for other types of organ fibrosis.

Although many transcription factors have been reported to regulate Wnt5a expression [25, 26], we confirmed that Egr-1 regulated Wnt5a expression in human oral fibroblasts. Interestingly, Egr-1 is a typical immediate early gene (IEG) [38]. IEGs are genes which are activated transiently and rapidly in response to a wide variety of cellular stimuli. These characteristics of Egr-1 are consistent with the fluctuations in arecoline concentration in the saliva of areca nut chewers [24]. In this study, we reproduced this fluctuation in arecoline concentration by replacing half of the cell culture medium every 24 h. Our findings showed that MMA or CHA treatment could block the effect of arecoline treatment on fibroblast proliferation. MMA is a U.S. Food and Drug Administration-approved drug that is used to treat fibrosis, cancer, and neurodegenerative diseases [39–41]. Therefore, additional exploration of its mechanisms in additional disease models may indicate that MMA is a promising drug candidate for the treatment of OSF and oral cancer.

We observed that small doses of arecoline can stimulate fibroblast proliferation in a short time; however, in reality, people need to chew areca nuts for a long time to develop OSF. In vivo, high doses of arecoline often lead to oral inflammation. Studies have shown that chewing betel nuts regularly can promote the expression of pro-inflammatory mediators, and provide an oral microenvironment with pro-inflammatory function that promotes the occurrence of cancer [42]. Arecoline has been shown to induce ROS in different cell types [43], and the activation of NF-κ B may be the basis of ROS production in this context [44]. Therefore, chewing betel nuts may cause oxidative stress, induce the expression of inflammatory factors, and prolong inflammation. Arecoline is also known to be somewhat immunosuppressive. Chang et al. found that areca nut extracts could promote the secretion of COX-2, IL-1α, and PGE2, resulting in suppression of the immune system [42]. Chang et al. also reported that areca nut extracts can promote the increase of lipopolysaccharide in the innate immune response, thus inhibiting the recovery of white blood cells and further affecting immune cell function [45]. However, this study only shows the effects of arecoline on oral fibroblasts cultured in vitro. A better understanding of the comprehensive effects of chewing areca nuts on oral health requires more in-depth and systematic cellular and animal studies.

## Conclusions

This study found that high doses of arecoline could inhibit human oral fibroblast proliferation, while low doses of arecoline could promote the proliferation of human oral fibroblasts. This study further determined that Egr-1 mediates the effects of low-dose arecoline treatment on human oral mucosa fibroblast proliferation by transactivating the expression of Wnt5a. The findings of this study indicate that Egr inhibitors and Wnt5a antibodies are potential therapies for treatment of OSF and oral cancer.

## Methods

### Reagents

Arecoline (#S2614, Selleck Chemicals), Recombinant human Wnt1 protein (#ab84080, Abcam plc.), Recombinant human Wnt2 protein (#H00007472-P01, Bio-Techne China Co. Ltd.), Recombinant human Wnt5a protein (#645-WN-010, R&D system, Wiesbaden-Nordenstadt, Germany), Mithramycin A (Sigma), Chromomycin A3 (Sigma) and antibodies were used at the indicated concentrations and time points. Lipofectamine LTX (#15338100, Invitrogen) and Lipofectamine RNAiMAX (#13778150, Invitrogen) were used for transient gene or siRNA transfection of cells. The following primary antibodies were used: Wnt1 (#ab15251), Wnt2 (#ab109222), Wnt5a (#ab179824) and GAPDH (#ab181602) were from Abcam plc..

### Cell culture and treatment

Human oral fibroblasts (ATCC® PCS-201-018™) were cultured in fibroblast basal medium (ATCC PCS201030) containing 2% heated-inactivated fetal bovine serum supplemented with 5 ng/ml rh FGF-b, 7.5 mM L-glutamine, 50 μg/ml Ascorbic acid, 1 μg/ml Hydrocortisone Hemisuccinate, 5 μg/ml rh Insulin, 1 mM sodium pyruvate, 50 μM 2-mercaptoethanol, 10 Units/ml penicillin, 25 μg/ml Amphotericin B and 10 μg/ml streptomycin.

### DNA growth assay

Following treatment of cells, the media was discarded, cells were solubilized for 30 min at 37 °C in 0.1% SDS and the amount of DNA was estimated using a Hoechst 33258 microassay [46].

### Immunofluorescence (IF) assay

IF assay was performed as described previously [47]. After treatment, cells were fixed using freshly prepared 4% paraformaldehyde, followed by permeabilization with 0.1% Triton X-100 in TBS and blocking in 3% donkey serum. Then, cells were subjected to IF assay with PCNA antibody (1:500; Abcam Cat#ab92552). PCNA antibody were detected with an anti-Rabbit secondary antibody conjugated to Alexa Fluor 555 (Abcam Cat#ab150062). The nuclei were stained with Hoechst 33258. Microscopy was performed using an Olympus Fluoview confocal microscope.

### Real-time polymerase chain reaction (real-time PCR)

Total RNA was extracted using the EastepTM Universal RNA Extraction Kit (Promega, Madison, WI) and transversely transcribed with the GoScriptTM Reverse Transcription System (Promega, Madison, WI) according to the manufacturer’s instruction. Real-time PCR was performed in triplicate with GoTaq® qPCR Master Mix (Promega, Madison, WI) and run on the ABI 7500 Fast Real-Time PCR System (Applied Biosystems, Framingham, MA). The mRNA level of the housekeeping gene *β-actin* was used as a control. The following primer pairs were used: Wnt1 forward (5′- GAA ATG CCC CCA TTC TCC CA − 3′) and reverse (5′- CGT GGC TCT GTA TCC ACG TT − 3′); Wnt2 forward (5′- GGA TGA CCA AGT GTG GGT GT − 3′) and reverse (5′- GGT CAT GTA GCG GTT GTC CA − 3′); Wnt2b forward (5′- GAC GGC AGT ACC TGG CAT AC − 3′) and reverse (5′- TGT CAC AGA TCA CTC GTG CC − 3′); Wnt3 forward (5′- ACT TTT GTG AGC CCA ACC CA − 3′) and reverse (5′- TTC TCC GTC CTC GTG TTG TG − 3′); Wnt3a forward (5′- AGC AGG ACT CCC ACC TAA AC − 3′) and reverse (5′- AGA GGA GAC ACT AGC TCC AGG − 3′); Wnt4 forward (5′- CAT GAG TCC CCG CTC GTG-3′) and reverse (5′- CCA GGT ACA GCC AGT TGC TC − 3′); Wnt4 forward (5′- CAT GAG TCC CCG CTC GTG-3′) and reverse (5′- CCA GGT ACA GCC AGT TGC TC − 3′); Wnt5a forward (5′- CTC CAT TCC TGG GCG CAT C-3′) and reverse (5′- GCA GTG AAC CGG AGC TGA AG − 3′); Wnt5b forward (5′- AGC CAC AGT GAC CAT TAG CAG-3′) and reverse (5′- AGT AGG GTT CCC TCT GTC ACC − 3′); Wnt6 forward (5′- TGG CCT CTA GGA GGA AAC AGT-3′) and reverse (5′- ATT GAT ACT AAC CTC ACC CAC C − 3′); Wnt7a forward (5′- ACG GCC TGT GCT TCT TCT TA-3′) and reverse (5′- GCC CAC TTG GCA AAC AGA AC − 3′); Wnt7b forward (5′- AAG TGC GGA CAC ATT GGC − 3′) and reverse (5′- ACC TCG AAG CCC GGT TGA − 3′); Wnt8a forward (5′- AAG AGC TGC TGA TTT CCT CCC − 3′) and reverse (5′- AGG GCC AAG TCC AGA GAA GT − 3′); Wnt8b forward (5′- ACA GCT GGT CGG TGA ACA AT − 3′) and reverse (5′- CTG CCA CAC TGC TGG AGT AA − 3′); Wnt9a forward (5′- GGC AAG ATG CTG GAT GGG T − 3′) and reverse (5′- GGT CGC AGG CCT TGT AGT G − 3′); Wnt9b forward (5′- GAG ATG CTA GAG GGC GCA G − 3′) and reverse (5′- CAG TGC CCA ATC CTG GGA AG − 3′); Wnt10a forward (5′- CTG GGT GCT CCT GTT CTT CC − 3′) and reverse (5′- TTA GGC ACA CTG TGT TGG CA − 3′); Wnt10b forward (5′- CTG ACA AGG GGA CAG AAC CC − 3′) and reverse (5′- CAG GAC CTC CAG TGG TTT GG − 3′); Wnt11 forward (5′- GGG GTG GCA CTT CTC AAT TC − 3′) and reverse (5′- TGC CGA GTT CAC TTG ACG A − 3′); Wnt16 forward (5′- TAC AGC TCC CTG CAA ACG AG − 3′) and reverse (5′- CCA AGT TAT CCC TCG CCC TC -3′); GAPDH forward (5′- GAC AGT CAG CCG CAT CTT CT − 3′) and reverse (5′- GCG CCC AAT ACG ACC AAA TC − 3′).

### Western blotting

Proteins from cells (30 μg) were separated by SDS-PAGE and transferred onto PVDF membranes. Then the members were blotted with primary antibodies at 4 °C overnight. Blots were incubated with HRP-conjugated secondary antibody for 1 h. The proteins were visualized using the ECL Plus WB detection system (Pierce, Rockford, IL).

### Constructs

The Human Wnt5a-luciferase (pGL3-Wnt5a, containing nucleotides from − 2152 to + 275 of the Human Wnt5a gene (Gene ID: 7474) reporter were cloned into the pGL3-basic vector. Constructs were transfected into cells using Lipofectamine LTX.

### Dual-luciferase reporter assays

Constructs were transfected into cells using Lipofectamine LTX. For the dual-luciferase reporter assays, cells were transfected with 1 μg of a luciferase reporter plasmid and 200 ng of the pRL-CMV Renilla luciferase reporter plasmid (Promega). After transfection, cells were kept in conditioned media for 12 or 24 h and then transferred to treatment media for 12 h. Firefly luciferase activity was normalized to Renilla luciferase activity according to the protocol.

### siRNA interference

Wnt1, Wnt2, Wnt5a, NF-κB p65, Smad2, Smad3 or Egr-1 specific siRNAs were from Dharmacon (ONTARGETplus SMARTpool, named as si-1) and Santacruz (named as si-2); the negative control (NC) siRNA (no silencing small RNA fragment) was synthesized by GenChem Co. (Shanghai, China). siRNAs were transfected into cells using Lipofectamine RNAiMAX transfection reagent.

### Statistical analysis

Data are presented as mean ± SEM. Statistical analyses were performed with GraphPad Prism 6 (GraphPad Software, La Jolla, CA, USA) using ANOVA followed by post hoc tests as appropriate. Statistical significance was declared when *p* < 0.05. The experimenters were not blind to group assignment and no data were omitted.

## Supplementary Information

**Additional file 1.**

**Additional file 2.**

**Additional file 3.**

**Additional file 4.**

## Data Availability

The datasets analyzed during the current study are available from the corresponding author on request.

## Abbreviations

Wnt5a: Wingless-type family member 5a
Egr-1: Early growth response protein 1
NF-κB: Nuclear factor kappa-B
FOXO1: Forkhead box O1
Smad: Sma and Mad proteins
TGF: Transforming growth factor
OSF: Oral submucosal fibrosis
