# Supplementary figures and images for: Egr-1 mediates low-dose arecoline induced human oral mucosa fibroblast proliferation via transactivation of Wnt5a expression
Source: BMC Mol Cell Biol. 2020 Nov 10;21:80. doi: 10.1186/s12860-020-00325-7 (PMC7653895; doi:10.1186/s12860-020-00325-7)

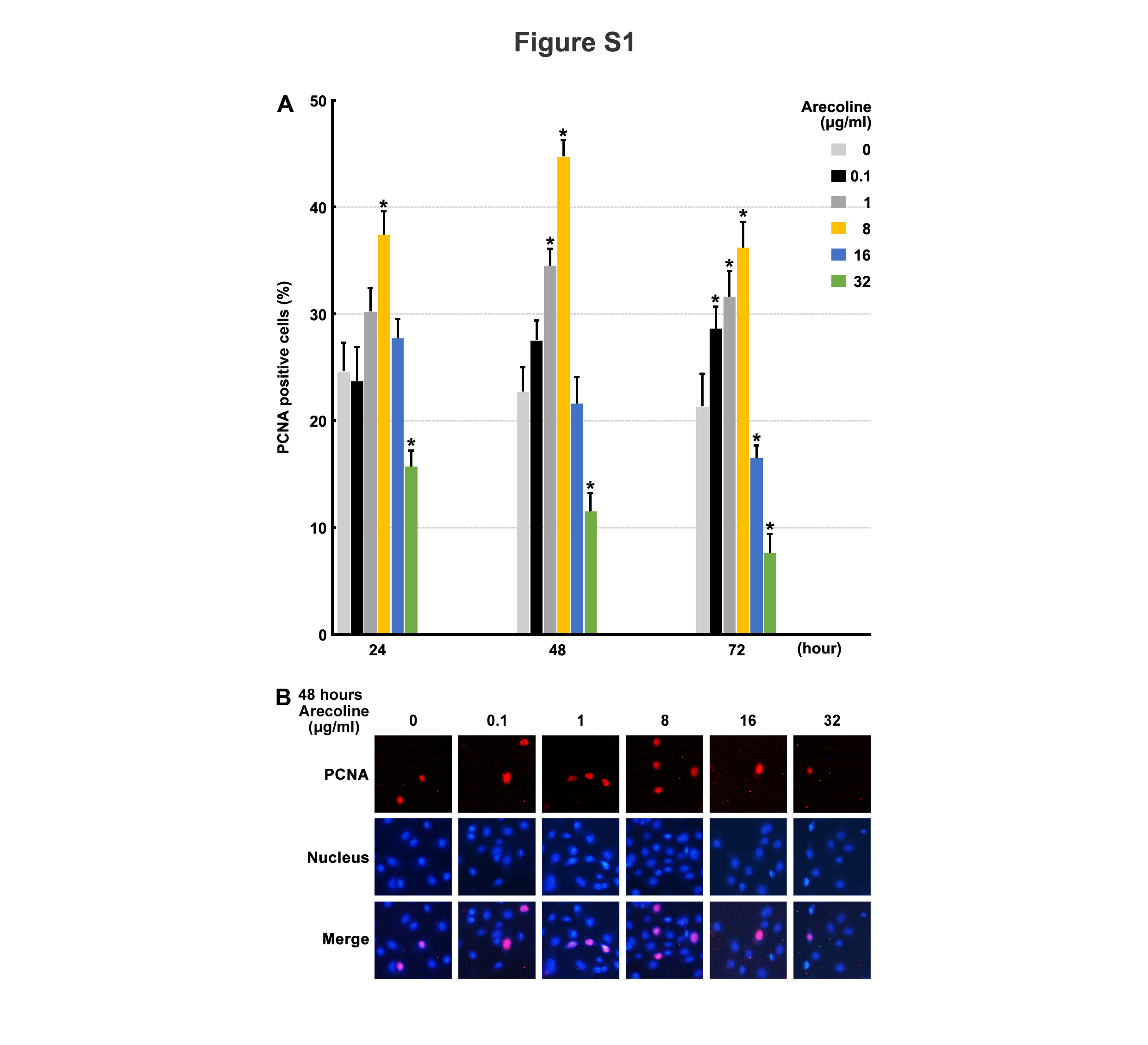

Supplement: Supplementary file 1 — Additional file 1. [file 12860_2020_325_MOESM1_ESM.jpg]

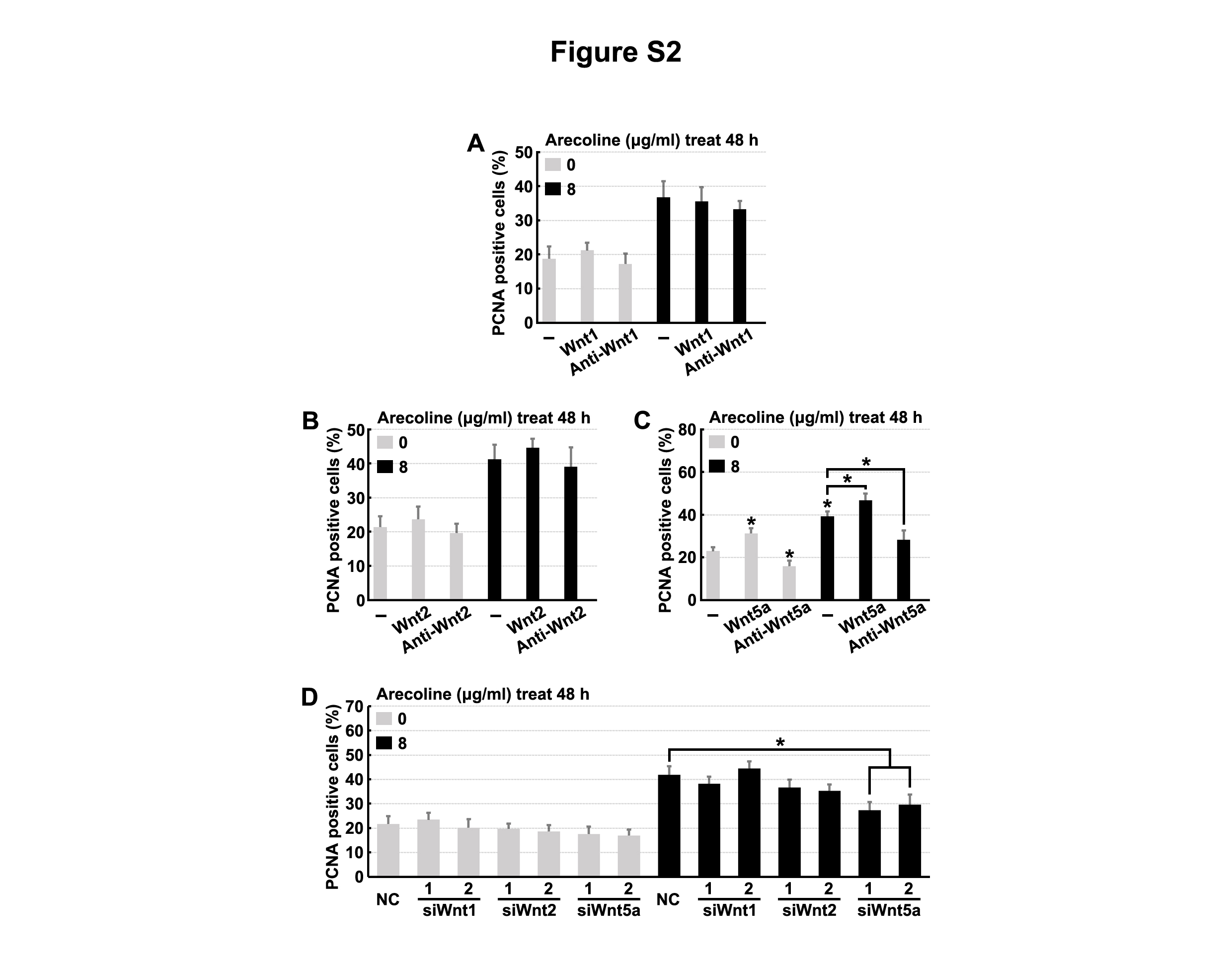

Supplement: Supplementary file 2 — Additional file 2. [file 12860_2020_325_MOESM2_ESM.jpg]

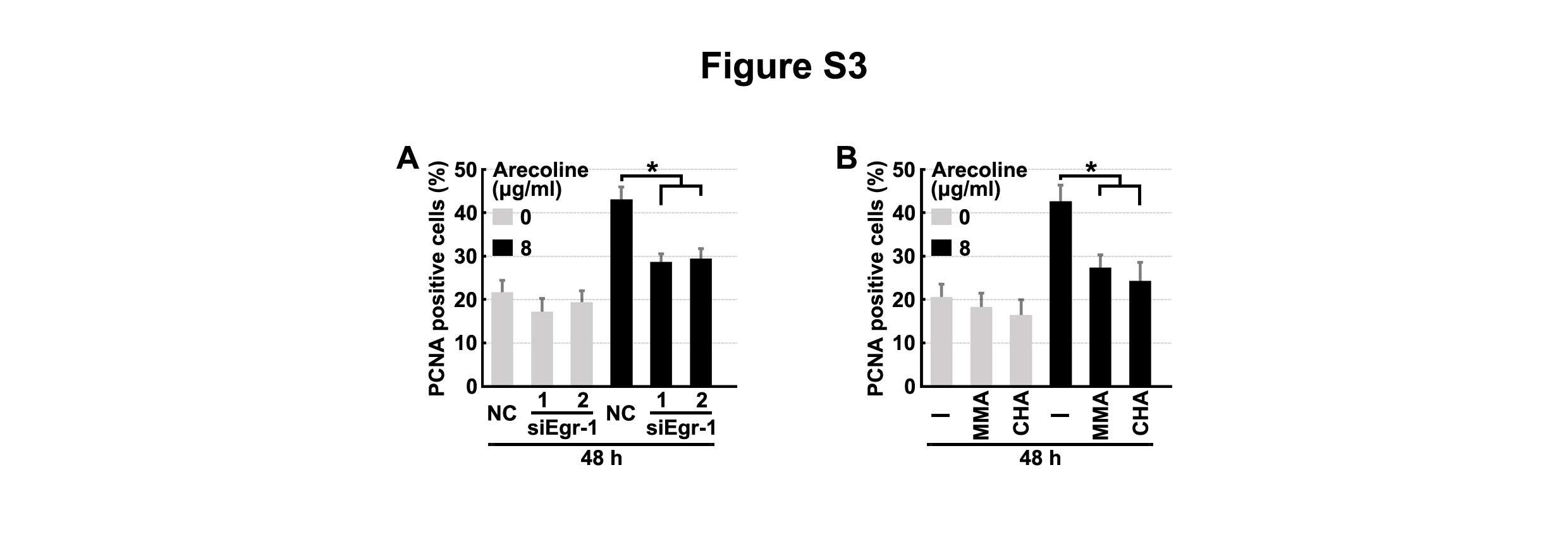

Supplement: Supplementary file 3 — Additional file 3. [file 12860_2020_325_MOESM3_ESM.jpg]
