# Supplementary material for: Egr-1 mediates low-dose arecoline induced human oral mucosa fibroblast proliferation via transactivation of Wnt5a expression
Source: BMC Mol Cell Biol. 2020 Nov 10;21:80. doi: 10.1186/s12860-020-00325-7 (PMC7653895; doi:10.1186/s12860-020-00325-7)
Supplement: Supplementary file 4 — Additional file 4. [file 12860_2020_325_MOESM4_ESM.docx]

**Figure 2A line 1 Wnt1**

**
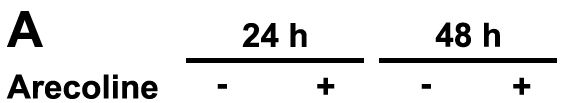
**


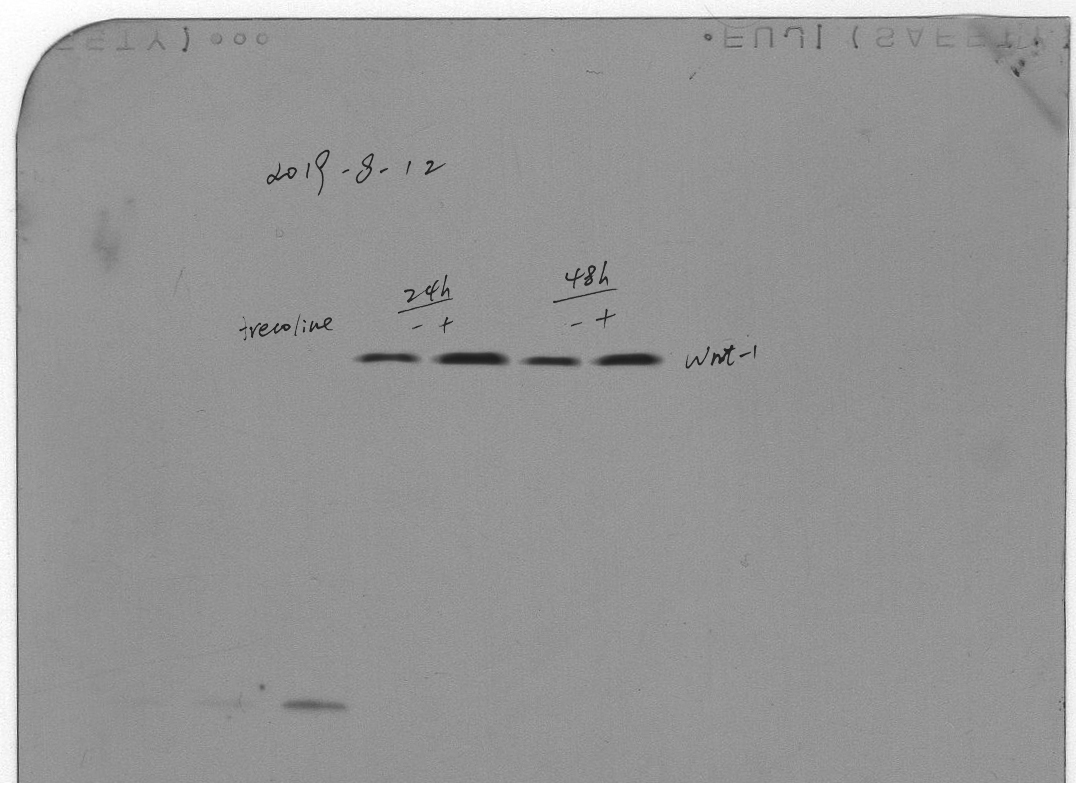


**Wnt1**

**Fig. 2** Arecoline promotes fibroblasts proliferation by inducing Wnt5a expression. **a.** Human oral fibroblasts were treated with or without 8 μg/ml Arecoline for indicated times, then cell lysates were analyzed by Western blotting using indicated antibodies.

**Figure 2A line 2 Wnt2**

**
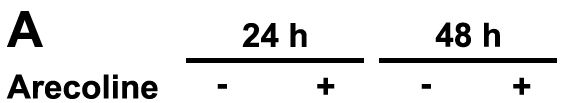
**


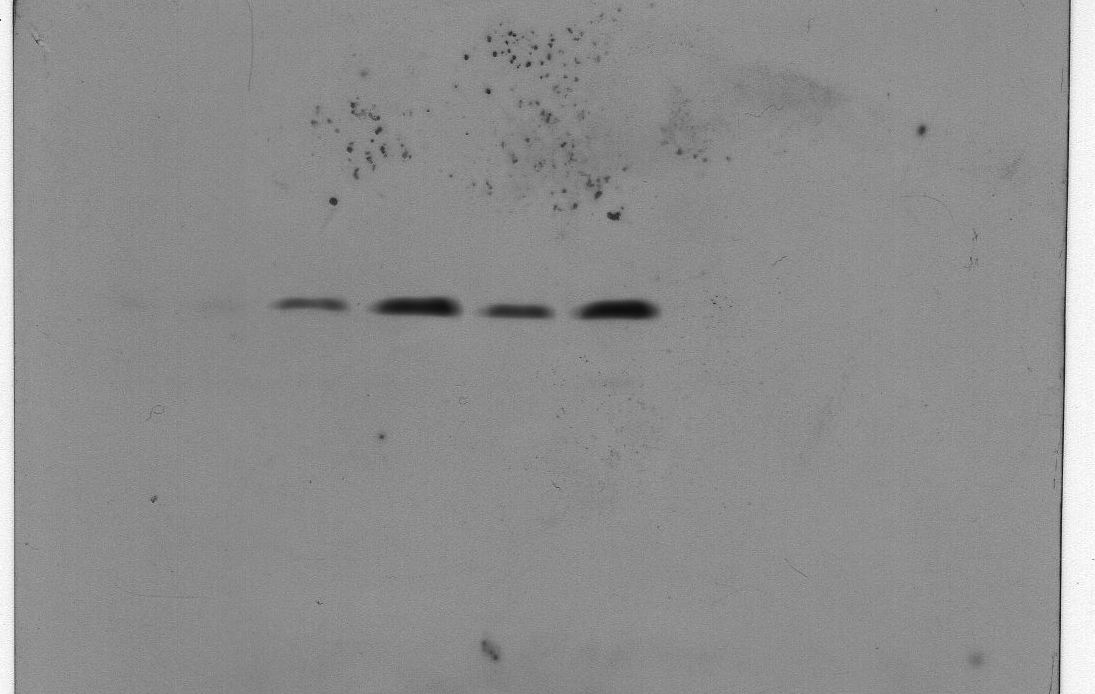


**Wnt2**

**Fig. 2** Arecoline promotes fibroblasts proliferation by inducing Wnt5a expression. **a.** Human oral fibroblasts were treated with or without 8 μg/ml Arecoline for indicated times, then cell lysates were analyzed by Western blotting using indicated antibodies.

**
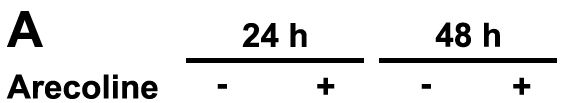
Figure 2A line 3 Wnt5a**


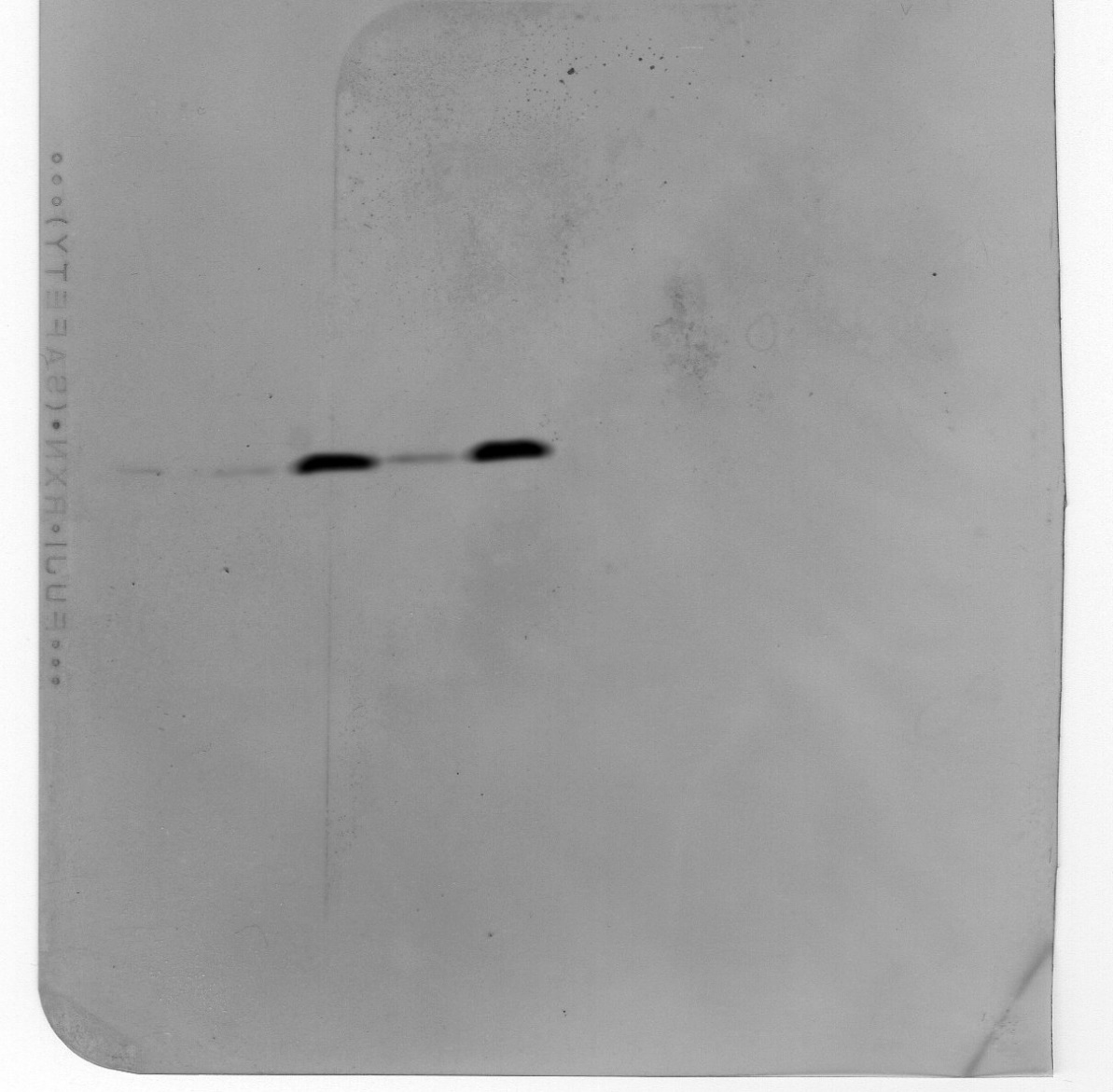


**Wnt5a**

**Fig. 2** Arecoline promotes fibroblasts proliferation by inducing Wnt5a expression. **a.** Human oral fibroblasts were treated with or without 8 μg/ml Arecoline for indicated times, then cell lysates were analyzed by Western blotting using indicated antibodies.

**Figure 2A line 4 GAPDH**

**
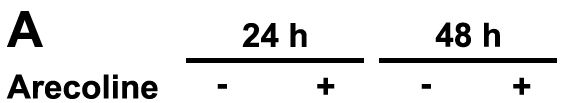
**


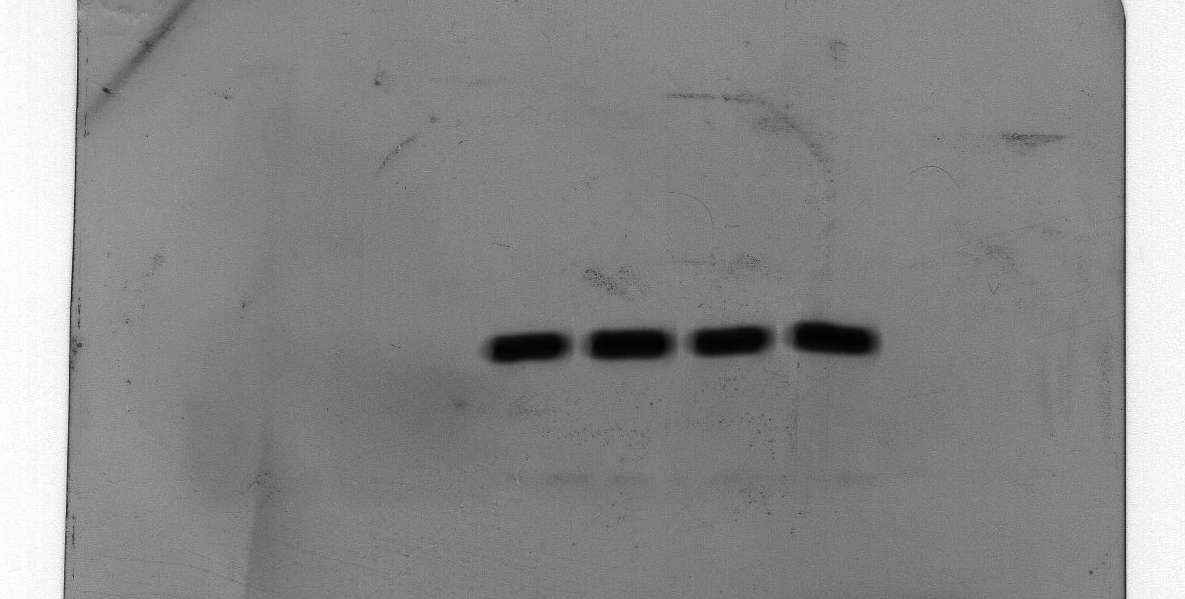


**GAPDH**

**Fig. 2** Arecoline promotes fibroblasts proliferation by inducing Wnt5a expression. **a.** Human oral fibroblasts were treated with or without 8 μg/ml Arecoline for indicated times, then cell lysates were analyzed by Western blotting using indicated antibodies.

**Figure 3C line 1 Wnt5a**

**
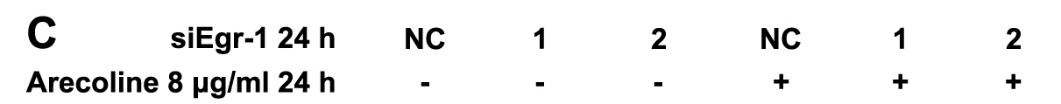
**

**
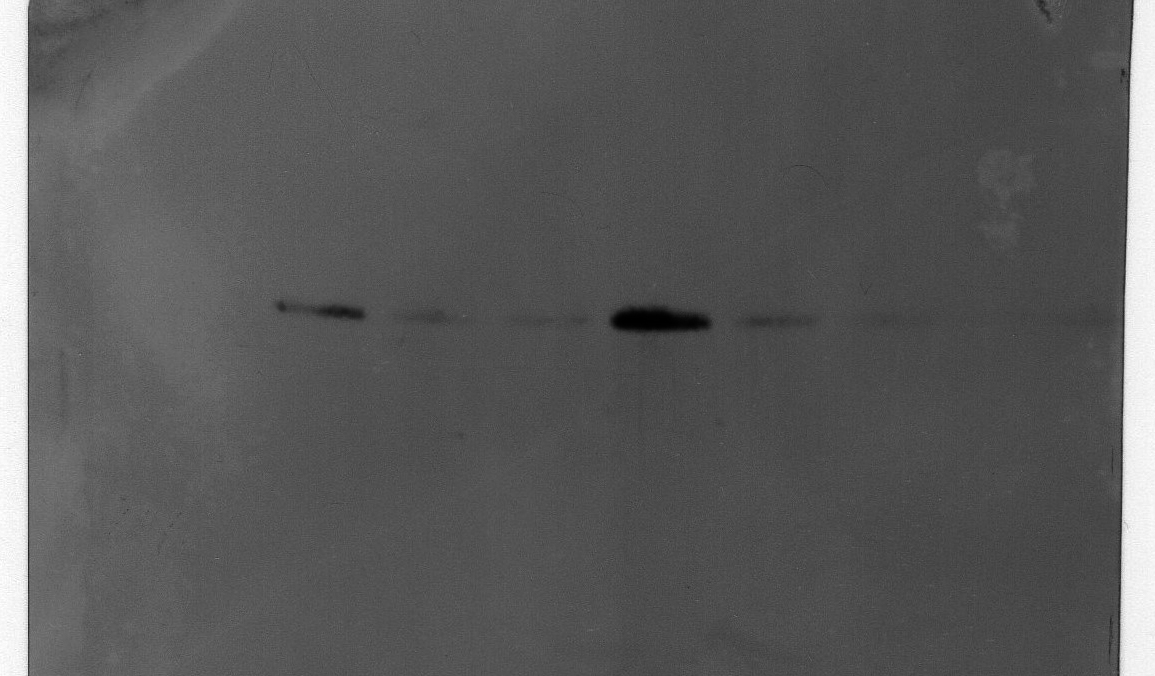
**

**Wnt5a**

**Fig. 3** Egr-1 is necessary for the expression of Wnt5a. **c.** Human oral fibroblasts were treated with 8 μg/ml Arecoline and Egr-1 siRNAs for 24 h, then cell lysates were analyzed by Western blotting using indicated antibodies.

**Figure 3C line 2 GAPDH**

**
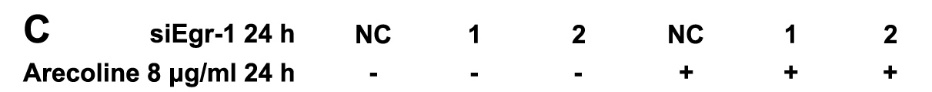
**

**
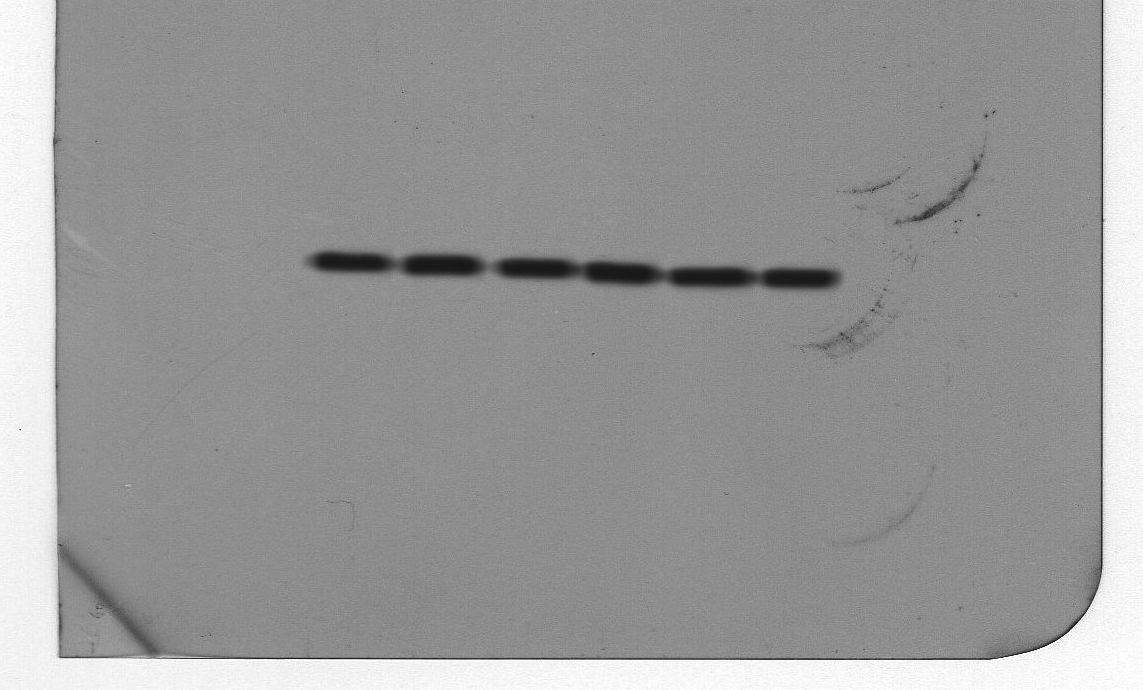
**

**GAPDH**

**Fig. 3** Egr-1 is necessary for the expression of Wnt5a. **c.** Human oral fibroblasts were treated with 8 μg/ml Arecoline and Egr-1 siRNAs for 24 h, then cell lysates were analyzed by Western blotting using indicated antibodies.
